# The effect of a topical combination of clonidine and pentoxifylline on post-traumatic neuropathic pain patients: study protocol for a randomized, double-blind placebo-controlled trial

**DOI:** 10.1186/s13063-021-05088-w

**Published:** 2021-02-17

**Authors:** Oli Abate Fulas, André Laferrière, D. Mark A. Ware, Yoram Shir, Terence J. Coderre

**Affiliations:** 1 Department of Anesthesia, McGill University, 3655 Promenade Sir William Osler, Montreal, QC H3G 1Y6 Canada; 2 Alan Edwards Pain Management Unit, McGill University Health Centre, 1650 Cedar Avenue, Montreal, QC H3G 1A4 Canada

**Keywords:** Topical treatment, Post-traumatic peripheral neuropathic pain, Pentoxifylline, Clonidine, Dynamic mechanical allodynia, Punctate hyperalgesia

## Abstract

**Background:**

First-line pharmacotherapy for neuropathic pain entails the use of systemic antidepressants and anticonvulsants. These drugs are not optimally effective and poorly tolerated, especially for older patients with comorbid conditions. Given the high number of such patients, there is a need for a greater repertoire of safer and more effective analgesics. Clonidine and pentoxifylline are vasodilator agents that work synergistically to enhance tissue perfusion and oxygenation. The topical administration of these drugs, individually and in combination, has shown anti-nociceptive properties in rodent models of neuropathic pain. A topically-administered combination of clonidine and pentoxifylline also effectively reduced the intensity of both spontaneous and evoked pain in healthy volunteers with experimentally-induced neuropathic pain. The next step in advancing this formulation to clinical use is the undertaking of a phase II clinical study to assess its efficacy and safety in neuropathic pain patients.

**Methods/design:**

This is a study protocol for a randomized, double-blind, placebo-controlled, phase II clinical trial with a cross-over design. It is a single-centered, 5-week study that will enroll a total of 32 patients with post-traumatic peripheral neuropathic pain. Patients will be treated topically with either a combination of clonidine and pentoxifylline or placebo for a period of 2 weeks each, in randomly assigned order across patients, with an intervening washout period of 1 week. The primary outcome measures of the study are the intensity of spontaneous pain recorded daily in a pain diary with a visual analog scale, and the degree of mechanical allodynia evoked by a brush stimulus. The secondary outcome measures of the study include scores of pain relief and change in the area of punctate hyperalgesia. This trial has been prospectively registered with ClinicalTrials.gov on November 1, 2017. ClinicalTrials.gov Identifier: NCT03342950.

**Discussion:**

The analgesic use of topical treatment with clonidine and pentoxifylline in combination has not been investigated in post-traumatic neuropathic pain. This study could generate the first evidence for the efficacy and safety of the formulation in alleviating pain in patients with neuropathic pain. Furthermore, this trial will provide objective grounds for the investigation of other agents that enhance tissue oxygenation in the topical treatment of peripheral neuropathic pain.

**Trial registration:**

This trial has been registered with ClinicalTrials.gov owned by NIH’s US National Library of Medicine.

ClinicalTrials.gov NCT03342950. Registered on November 1, 2017 (trial was prospectively registered).

**Protocol version and identifiers:**

This is protocol version 5, dated June 2018. McGill University Health Center (MUHC) Reaseach Ethics Board (REB) identification number: TTNP 2018-3906.

## Background

Peripheral neuropathic pain is a chronic pain condition that is a direct outcome of damage to the peripheral nerves [1]. The damage could result from a myriad of etiologies like physical trauma, metabolic dysfunctions like diabetes mellitus, or toxic effects of chemotherapeutic agents [2]. The patients suffer from debilitating pain of burning, tingling, or electric shock-like quality, along with hypersensitivity to touch, pressure, and changes in temperature [2]. Although the pathophysiology of peripheral neuropathic pain involves an interplay between neuronal, immune, neuroimmune, vascular, and metabolic factors, first-line pharmacologic analgesics in clinical use are mostly antidepressants and anticonvulsants that solely target modulatory processes in the central nervous system [3]. In addition to being sub-optimally therapeutic, these agents have significant side effects often making them intolerable especially to patients of older age and co-morbid conditions [4, 5]. There is an indisputable need for effective and safe analgesic options for patients with peripheral neuropathic pain.

Peripheral neuropathic pain of varying etiologies involves microvascular changes that result in ischemic hypoxia of the affected nerves. Functionally, deficits have been reported in the endothelial production of vasodilatory nitric oxide (NO), which normally acts to counteract the basal vasoconstrictive sympathetic tone [6]. Structural changes to the endoneurial vessels have also been observed, including endothelial cell layer swelling and capillary basement membrane thickening [7, 8]. These microvascular changes impair endoneurial nutritive perfusion and oxygenation, which can directly impact the excitability of the peripheral nerves [9]. The tactile allodynia and thermal hyperalgesia exhibited by neuropathic rats are significantly correlated to the extent of endoneurial hypoxia in the affected peripheral nerves [10, 11]. Therefore, treatments aimed at enhancing tissue oxygenation by increasing arterial and capillary flow may effectively relieve symptoms in patients with neuropathic pain.

Microvascular perfusion is under the regulation of vasodilatory molecules produced by the endothelium like NO and the vasoconstrictive transmitter norepinephrine, which is released from sympathetic post-ganglionic neurons [12, 13]. NO’s vasodilator effect occurs by the augmentation of vascular smooth muscle cyclic mononucleotide level that in turn inhibits calcium influx and smooth muscle contraction, a mechanism that is counterbalanced by the activity of phosphodiesterase enzymes that degrade cyclic mononucleotides [14]. Drugs with phosphodiesterase inhibitor activity, including pentoxifylline, produce vasodilation and enhance blood flow [15, 16]. Primarily indicated for peripheral arterial disease, pentoxifylline inhibits a broad isoform of phosphodiesterase enzymes resulting in enhanced blood flow, reduced platelet aggregation, decreased blood viscosity, and increased red blood cell flexibility, all of which aid in alleviating poor microvascular perfusion [16–18]. On the other hand, microvascular perfusion is further degraded by the vasoconstrictive effect of sympathetic activity when norepinephrine binds to α-adrenergic receptors on vascular smooth muscle cells [19]. Clonidine is a drug that counteracts sympathetic vasoconstriction by activating presynaptic α_2_-adrenergic receptors and reducing the release of norepinephrine both centrally and at peripheral sympathetic nerve endings [20]. It has also been reported to activate endothelial α_2_-receptors resulting in increased release of NO and subsequent vasodilation [21]. Therefore, clonidine and pentoxifylline act via complementary mechanisms to increase microvascular caliber, blood flow, and tissue perfusion.

In neuropathic pain, the effects of microvascular dysfunction are most adverse on the peripheral nerve’s distal endings in the skin, also called intraepidermal nerve fibers [22, 23]. Thus, topical microvascular-targeted local and cutaneous interventions that are effective in combating poor nutritive perfusion of the peripheral nerve endings are beneficial. The topical route allows the delivery of effective drugs in high concentrations at the local target site while avoiding undesired systemic and adverse effects [24]. Prior studies by our group have demonstrated that topical combination of α-adrenergic receptor agonists and phosphodiesterase inhibitors effectively alleviate tactile allodynia and microvascular dysfunction in rodent models of post-traumatic, diabetic, and chemotherapy-induced neuropathic pain [25]. α_2_-Adrenergic receptor agonists have also been used topically to alleviate chronic pain in humans [26, 27]. Moreover, as an initial step in determining the translatability of topical combination of clonidine and pentoxifylline as analgesics, we have tested the effect of the formulation in healthy volunteers in an experimentally-induced surrogate for neuropathic pain using a double-blind randomized and controlled design [28]. In the study, an experimental model of neuropathic pain was induced by tourniquet exposure following intradermal capsaicin injection. Subsequent topical treatment with the combination of clonidine and pentoxifylline significantly reduced the superficial and deep “neuropathic-like” pain reported by the volunteers. Moreover, areas of dynamic allodynia and mechanical hyperalgesia were significantly reduced to an extent that exceeded the published effectiveness of most analgesics, including opioids and gabapentinoids [29, 30]. The current protocol aims to examine the second translational investigative level to determine the effectiveness and safety of the same topical combination of clonidine and pentoxifylline in relieving pain in patients with post-traumatic neuropathic pain.

## Objectives

The main focus of this phase II proof-of-concept clinical trial is to provide preliminary evidence for the analgesic efficacy and safety of the topical combination of clonidine and pentoxifylline on post-traumatic neropathic pain using a small and well-defined patient population. More specifically, the study will measure the magnitude of relief of spontaneous pain produced by the treatment using daily recordings of pain level with visual analog scale (VAS)-based pain diaries. To estimate the effect of the treatment on evoked pain, measures for severity of dynamic mechanical hyperalgesia and area of punctate hyperalgesia will also be assessed.

## Methods

### Trial design

**Fig. 1:**
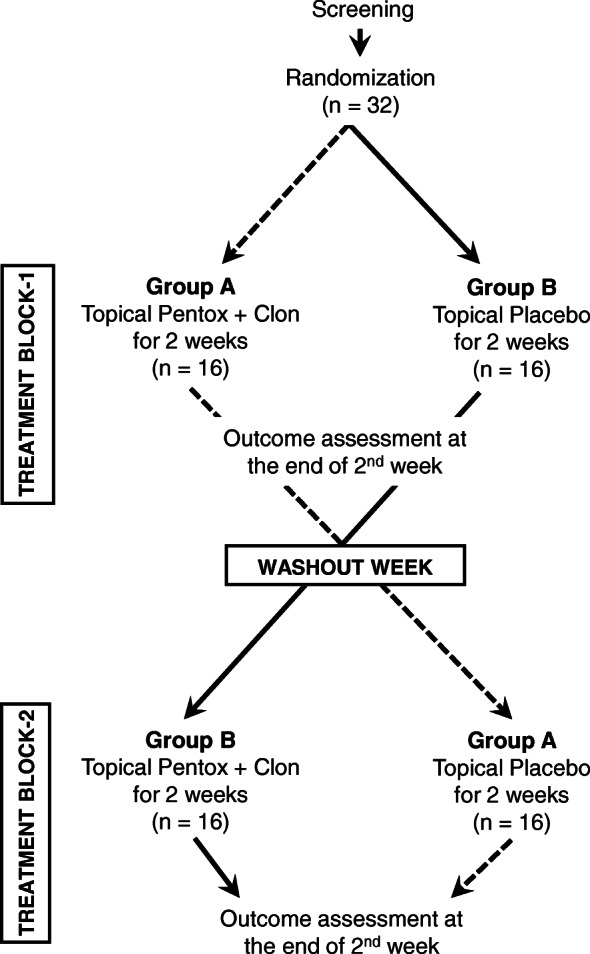
Study flow chart

This trial uses a randomized, double-blind, placebo-controlled cross-over study design. In this counterbalanced cross-over study, enrolled patients will be treated with active drug and placebo for periods of 2 weeks each, with an intervening 1-week washout period (Fig. 1).

### Study setting

This is a single-center study taking place in the Alan Edwards Pain Management Unit at the Montreal General Hospital, Montreal, Quebec. The study has been authorized by the McGill University Health Centre Research Ethics Board (TTNP: 2018-3906) and Health Canada (control number: 201880). Moreover, it is registered with NIH’s US National Library of Medicine database: ClinicalTrials.gov (Identifier: NCT03342950).

### Study process

A summary of the study process is outlined in Table 1. The first screening visit will entail obtaining informed consent; reviewing patients’ medical history, including the patients’ current medications; performing sensory tests for mechanical sensitivity; collecting blood samples for liver and kidney function; and obtaining urine samples from pre-menopausal women for a pregnancy test. Patients will be provided with a pain diary to record their daily pain intensity for the 7 days that follow the screening visit. Patients with normal laboratory test results of liver and kidney function will be contacted over the phone. After the assessment of pain diaries for pain intensity, patients that meet the eligibility requirements of the study (see below) will be provided with study drugs according to the treatment regimen assigned for the first 2 weeks of the trial. Regular medications used before enrolment into the study will be continued.

**Table 1:**
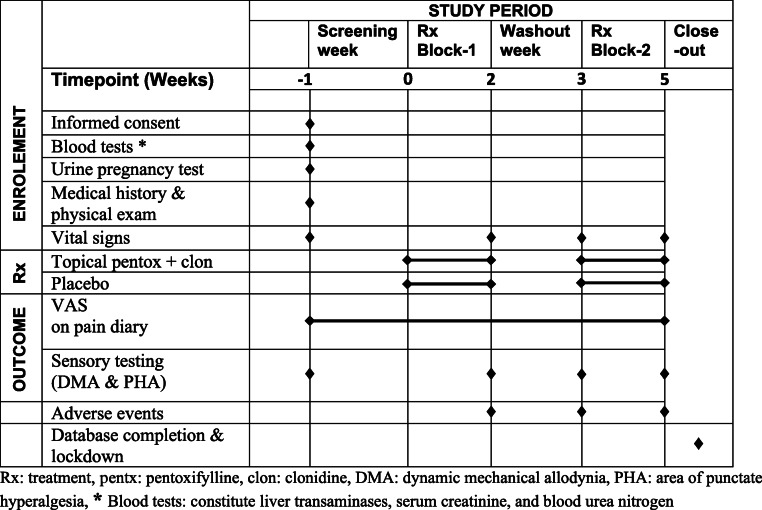
Study process

Throughout each treatment period and during the washout, patients will be asked to record their average pain scores in a pain diary, once daily in the evening. At the beginning and end of each treatment block, sensory testing will be performed to determine the degree of dynamic mechanical allodynia and area of punctate hyperalgesia. At the end of each treatment block, patients will be asked to score the degree of their overall pain relief. Adverse events will also be captured at the end of each treatment block and after the washout week.

### Eligibility criteria

Patients will have to fulfill all the following inclusion criteria:

1. Female or male patients, aged 18–70 years
2. An average spontaneous pain level of at least 4 on an 11-point numerical rating pain score (0 = no pain, 10 = worst pain possible) on at least 3 days during the week before the study
3. The existence of tactile allodynia, as a sign of neuropathic pain, following a traumatic peripheral nerve injury
4. Ability to communicate in English or French
5. Willing and able to sign an informed consent
6. Stable pain disease with no anticipated change in treatment for the next 5 weeks.
7. Female subjects of childbearing age must agree to use an effective method of contraception during the study period. Female subjects who utilize a hormonal contraceptive as one of their birth control methods must have consistently used the same method for at least 3 months before study drug dosing. Effective methods of contraception include double barrier methods [male condom (with spermicide), female condom (with spermicide), diaphragm with spermicide, cervical cap with spermicide or sponge with spermicide] or hormonal methods [oral contraceptives (either combined or progesterone only), injectable progesterone, subdermal contraceptive implant, transdermal contraceptive patch, contraceptive vaginal ring].

Any of the following conditions will exclude patients from this study:

1. Diabetes mellitus necessitating antihyperglycemic treatment or any other endocrine disease
2. Any liver disease, resulting in liver transaminase enzyme levels greater than three times the normal values
3. Chronic renal failure or kidney disease, resulting in serum creatinine levels > 133 μmol/l
4. Hypertension or taking of anti-hypertensive medication
5. Malignant disease or taking of chemotherapeutic agents
6. Known diagnosis of angina pectoris, arrhythmias, congestive heart failure, or peripheral arterial disease
7. Pregnancy or breastfeeding. Female patients of childbearing age must have a negative urine pregnancy test
8. Known allergic reaction to clonidine or pentoxifylline
9. Presence of a medical condition known to affect peripheral circulation (intermittent claudication, peripheral arterial disease, Raynaud’s syndrome)
10. Medication that interacts with clonidine or pentoxifylline [cardiovascular drugs such as angiotensin-converting enzyme inhibitors, alpha-blockers (prazosin, terazosin or doxazosin), beta-blockers (atenolol, metoprolol, propranolol), neuroleptics (butyrophenones, phenothiazines, thioxanthenes), calcium channel blockers (verapamil, diltiazem), and non-cardiovascular drugs such as diuretics, thyroxine, monoamine oxidase inhibitors, and selective serotonin reuptake inhibitors, as well as vitamin K antagonists and blood thinners like warfarin]
11. Any medical condition that might be impacted by clonidine or pentoxifylline, such as cardiovascular disease, cardiac rhythm disorders (sinus node dysfunction, atrial-ventricular blockade, or other conduction abnormalities), orthostatic regulation disturbances, and disorders of cerebral perfusion
12. A cerebral and/or retinal hemorrhage (in the last 5 years)

### Interventions

All participants will be randomized to treatment with either active drug or placebo in one of the two 2-week-long treatment blocks of the study. The active drug is an anhydrous topical solution containing 0.1% (W/V) clonidine and 5% (W/V) pentoxifylline. The placebo is a blank version of the anhydrous solution with constituents identical with the active drug except omitting the therapeutic agents, i.e., clonidine and pentoxifylline. The stability of the topical solutions at room temperature has been ascertained with mass spectrometric studies done upon the request of Health Canada. The exact composition of the active drug and placebo is as outlined in Table 2.

**Table 2:** Composition of 100 ml of the active drug topical solution with 0.1% (W/V) clonidine and 5% (W/V) pentoxifylline

**Solid ingredients**		**Amount (g)**
1	Clonidine	0.1
2	Pentoxifylline	5.0
**Liquid ingredients**		**Amount (ml)**
3	Anhydrous ethanol	6.2
4	Polyethylene glycol 400	18.9
5	Oleyl alcohol	50.8
6	Propylene glycol	18.9

Both the active drug and placebo solutions are colorless and will be packaged in identical Topi-Pump® dose-metered applicators that dispense 1 ml of topical solution with each pump. Participants will be instructed to apply one pump or 1 ml of the topical solution three times daily on an eight-hourly basis. They will also be asked to return the emptied pumps after use so adherence to treatment can be monitored.

### Outcomes

Primary outcome measures of the study are as follows:

1. The average daily pain intensity level is recorded by the patient at home in an REB approved pain diary that contains a visual analog scale (VAS) displaying the words “no pain” and “worst pain possible” on the left and right of a 100-mm line, respectively.
2. The degree of dynamic mechanical allodynia (DMA) is determined by stroking the most painfully sensitive area of the skin once at a velocity of 1–2 cm/s with a soft brush (bristle length by width: 20 × 15 mm). The patient then indicates the amount of pain evoked on an REB approved data collection sheet with a 100-mm VAS. This test will be stopped immediately at the request of the patient if it is too painful.

Secondary outcome measures of the study are as follows:

1. Pain relief scores are recorded on a 6-point categorical pain relief scale (0 worse pain to 5 complete pain relief).
2. Area of punctate hyperalgesia (PHA) is measured using punctate stimulation with a 26-g von Frey monofilament (Semmes Weinstein Monofilament Kit; Stoelting, Wood Dale, IL). Stimulation commences from outside the sensitive area toward the center of the sensitive area, along eight approximately evenly spaced radii, while asking the subject to report when the sensation becomes unpleasant or frankly painful. The points at which the sensations change will be marked and the markings gently transferred onto a lightweight transparent plastic sheet. The marked sheet is subsequently photographed with a ruler to calibrate the number of pixels per unit length during image analysis and area calculation.

### Sample size

Thirty-two patients will be recruited for this trial. This number of patients is chosen based on the assumption that during the active topical drug treatment period patients will experience a reduction of either of the primary outcome measures (spontaneous pain or intensity of DMA) of 20 mm or more on the 100 mm VAS (standard deviation assumed: 25, i.e., Cohen’s *d* = 0.8, alpha = 0.025 corrected for multiple testing; power = 95%). Based on a power calculation for two groups using a cross-over design, the estimated required sample size is 27, and we expect that a sample size of 32 is sufficient to detect changes in pain levels even if up to 18% of patients do not complete the study.

### Recruitment and informed consent

The thirty-two patients, females and males, with post-traumatic peripheral neuropathic pain will be recruited from the Quebec Pain Registry (more information available at www.quebecpainregistry.com) and the Alan Edwards Pain Management Unit at the Montreal General Hospital.

Informed consent is obtained by research study staff that have obtained Good Clinical Practice (GCP) clinical research training certification by the Research Institute of the McGill University Health Centre. Also, the research study staff will receive training on general standard operating procedures for clinical trials and specific tasks for conducting the study.

### Randomization and blinding

Randomization for treatment order (placebo–active drug/active drug–placebo) is performed by a coin flip done by a research coordinator assigned for this task who is not involved in patient interaction and outcome assessment. The same coordinator also ensures blinding by assigning a three-digit number code, from a computer-generated random list, to the Topi-Pumps used in each treatment block. The list of number codes and the identity of their corresponding treatment is only shared with the compounding pharmacy. The blinding applies to the patient, the study coordinator performing patient assessments, and the dispensing pharmacy.

### Implementation

The sequence of treatment for each patient will be determined based on the randomized treatment order assigned by the research coordinator described above. The enrolment and evaluation of eligible patients will be performed by two other research coordinators with the assistance of research nurses at the pain clinic. The two research coordinators and research nurses will be strictly blinded to the treatment allocations of the patients.

### Data collection, management, and confidentiality

Data will initially be collected on paper-based source documents filled out at every visit. Data will then be transferred promptly into a spreadsheet stored in a password protected drive. Data entry and management will be completed by one of the study coordinators and the sponsor will not have access to the data. All source documents and data sheets used in the clinical trial will not have patient identifying information; instead, patients will be assigned a study code number with the key securely stored to ensure confidentiality. All information pertaining to the study will be retained by the principal investigator for 25 years after completion as per guidelines of Health Canada.

### Auditing

The MUHC-REB will perform an annual review of the study activities and progress before issuing a renewal of authorization to resume the study. The review will require a detailed report of the number of recruited and enrolled participants; a tally of participants that completed, dropped out, or withdrew from the study with explanations; and a description of the nature and frequency of adverse events recorded during the study. Regular inspections and audits are expected to be performed by delegates from the MUHC-REB and Health Canada. The study coordinators will facilitate the process by granting access to all paper and electronic study records and data. The coordinators will also accompany the auditor on any tours and inspections of the study area.

### Statistical methods

Statistical analysis will be performed using an unpaired *t-*test to evaluate between-group differences in the 2-sequence groups, as is recommended for analyses in cross-over designs [31]. The summarized VAS pain scores during the second week of treatment will be compared between treatment groups adjusting for period and order. We will also use unpaired *t-*tests to assess carry-over effects by comparing baseline and washout period measures. DMA pain scores at the end of each treatment period will also be compared between treatment periods. Secondary analyses of pain relief and the area of punctate hyperalgesia will also be conducted using unpaired *t*-tests.

### Safety and adverse event outcomes

Both clonidine and pentoxifylline have a history of safe systemic use in humans, and there is evidence that topical application of these agents produces extremely low to undetectable plasma levels [27]. As no negative effects were observed in animals or adverse effects in our trial in healthy volunteers, we do not anticipate any adverse side-effects with our topical treatment [25, 28].

According to the FDA, rare events in patients taking pentoxifylline (although not established as caused by the drug) include anaphylactic reactions [32]. Subjects will be asked to immediately report any signs of anaphylaxis (itchy rash, lip or throat swelling) to the study coordinator, who will instruct the patient to go immediately to a hospital emergency room.

Risks involved in taking clonidine orally are dizziness, light-headedness, drowsiness, tiredness, and vision problems, which are caused by a decrease in blood pressure [33]. Patients will be asked to immediately report any of these symptoms to the study coordinator, who will instruct the patient to see the study physician, who will perform a medical evaluation, including assessment of blood pressure. If any subject’s systolic pressure is found to be below 80 mmHg, they will be asked to stop the treatments and will be removed from the study. Abrupt discontinuation of clonidine can occasionally result in a withdraw syndrome, characterized by a rebound increase in blood pressure and other symptoms and signs of sympathetic activity like restlessness, increased heart rate, and increased irritability [34]. This usually occurs after cessation of large oral doses and in some instances after transdermal treatment. There is minimal risk for this syndrome with our study drug, as the dose of clonidine used is much lower than oral or transdermal preparations. Patients will be asked to report to the study coordinator if any of these symptoms occur during the washout period of the study and the few days following completion of the study. Clinical evaluation and appropriate medical care will accordingly be provided.

All adverse events will be tabulated, summarized, and reviewed by the study physician. Serious adverse drug reactions, i.e., reactions causing hospitalization; persistent or significant disability; that is life-threatening or resulting in death, will be immediately reported to the study sponsor who is the primary investigator and the research ethics coordinator for the study site. Serious adverse drug reaction reporting will follow International Council for Harmonization (ICH) guidelines.

### Endpoints and post-trial care

Eligible patients are expected to have stable disease with no alteration to their treatment regimen in the 5 weeks preceding enrollment. If a patient develops uncontrolled pain with a clinical indication for a new treatment during the study period, the participation of the patient in the trial will be discontinued and the new treatment commenced. If any subject’s systolic pressure is found to drop below 80 mmHg during any section of the trial, they will be asked to stop the treatments and they will be removed from the study. Finally, in the event of an anaphylactic reaction to any of the topical treatments used in the study, the patient’s participation in the study will immediately be terminated. In both instances, the coordinator performing the randomization will send the study physician the information required to unblind him to the study drug the patient was taking so the information can be transferred to the medical team that will care for the patient.

Patients will be instructed to contact the study coordinator and/or physician if symptoms of clonidine withdrawal occur during washout or the week following completion of the study. In such an event, the study physician will perform a clinical evaluation of the patient and appropriate medical care will be provided. Unblinding and termination of participation in the study will depend on the study physician’s assessment of the patient’s clinical status.

### Compensation

Participants will receive reimbursement of travel expenses up to a sum of 100 Canadian dollars. If participants fail to complete the study, the compensation they receive will be pro-rated amounts based on the duration of participation.

## Discussion

The peripheral neuropathic pain patient population is composed of a sizable number of patients that can benefit from avoiding the systemic anticonvulsants and antidepressants that are first-line treatments of the condition [3–5]. Given their typically older age and comorbid conditions, these patients are at greater risk of developing adverse events from these recommended systemic treatments [4, 5]. This clinical study is a required step in developing a locally-administered, potentially effective and tolerable analgesic alternative for the treatment of peripheral neuropathic pain.

As a phase II clinical trial, this study is a proof-of-concept step to evaluate the potential efficacy and safety of the topical combination of clonidine and pentoxifylline in peripheral neuropathic pain [35]. To optimize the detection of hypothesized analgesic effects, the selection of the study population is restricted to a specific subgroup of neuropathic pain patients with an uncomplicated medical profile.

The cross-over design of the study was another step taken to increase the efficiency of the initial screening of the formulation’s analgesic effects. The design allows the use of a relatively small number of patients by minimizing between-subject data variability thereby reserving resources and time [35, 36]. We believe the 2-week duration of treatment is long enough to reveal the alleviation of neuropathic pain symptoms achieved. At the same time, the treatment is set to be brief enough to avoid long-term effects that can potentially change the underlying pathology preventing effective washout. The 1-week-long washout period is based on evidence obtained from the pharmacokinetics of constituent drugs and the length of analgesic effects measured from neuropathic rodent models and healthy volunteers treated with the formulation [25, 28, 33, 37]. It is expected that this time frame is sufficient in duration and carry-over effects are unlikely to occur.

Clinical trials with cross-over designs with relatively few patients have been used to successfully detect the efficacy of analgesics in painful diabetic neuropathy, post-herpetic neuralgia, and post-traumatic neuropathic pain [38–40]. The cross-over design assures the patients that treatment with both placebo and the active drug will occur prompting them to think more objectively about their pain levels in each period. This has resulted in significantly lower placebo group response rates in cross-over trials as compared to parallel-group trials, as reported in a meta-analysis of neuropathic pain trials [41].

With randomization and blinding, we have tried to maintain the internal validity of the study. Outcome measures were chosen to pointedly assess the analgesic efficacy and solely focus on discovering the degree of relief from spontaneous and evoked pain [42]. A broader investigation of physical-functional outcome and overall improvement of quality of life is undoubtedly necessary, but will be reserved for subsequent study phases that will be performed after initial proof of efficacy has been obtained for the formulation [43].

The outcome measures used as comparators in this study are kept the same as what has been used in the healthy volunteer human-study performed with the topical combination [28]. The measures comprehensively assess both spontaneous and evoked pain. The VAS ratings employed for assessments of daily levels of spontaneous pain and the degree of dynamic mechanical allodynia have a proven high degree of resolution and sensitivity in clinical pain research [44].

To the best of our knowledge, this study is the first randomized, double-blind, and placebo-controlled clinical trial to assess the analgesic efficacy of the topical combination of clonidine and pentoxifylline in neuropathic pain. Topical clonidine on its own has been investigated in this manner for the treatment of painful diabetic neuropathy and a positive outcome has been reported [27]. In complex regional pain syndrome, there have been clinical case reports of analgesic benefits achieved with the use of bare topical clonidine, and topical clonidine in combination with pentoxifylline, ketamine, and dimethyl sulfoxide [26, 45]. However, the level of evidence from these studies does not allow a reliable conclusion to be drawn on the therapeutic benefit of the topical treatments.

Lastly, relevant clinical data is required to affirm the abundant preclinical evidence on the role of poor nerve oxygenation and blood supply in the genesis of peripheral neuropathic pain [10, 25, 46–48]. This study is a means of achieving that, above and beyond presenting a specific drug formulation with potential efficacy for the treatment of peripheral neuropathic pain. The results can furthermore potentially promote the investigation of other vasodilatory agents for analgesic effects, building a new collection of safe and effective drugs for the treatment of peripheral neuropathic pain.

### Trial status

The study is currently actively recruiting and enrolling eligible participants. Recruitment started in January 2019 and the study is forecasted to be completed by January 2021. This is protocol version 5, dated June 2018. MUHC-REB identification number: TTNP 2018-3906. If significant changes requiring the modification of this protocol are required, the changes will be submitted for approval to the MUHC-REB through its web-based platform Nagano.

## Data Availability

There are no plans to publicly share the raw data or materials used during this clinical trial. There are plans to communicate the results of the analyzed data through conference presentations and publications in relevant journals.

## Abbreviations

clon: Clonidine
DMA: Dynamic mechanical allodynia
FDA: US Food and Drug Administration
GCP: Good Clinical Practice
IHC: International Council for Harmonization
MUHC: McGill University Health Centre
NO: Nitric oxide
pentox: Pentoxifylline
PHA: Punctate Hyperalgesia
REB: Research ethics board
VAS: Visual analog scale
W/V: Weight/volume
